# Supplementary material for: Analysis of PPARγ Signaling Activity in Psoriasis
Source: Int J Mol Sci. 2021 Aug 10;22(16):8603. doi: 10.3390/ijms22168603 (PMC8395241; doi:10.3390/ijms22168603)
Supplement: Supplementary file 1 [file ijms-22-08603-s001.zip › Supplemental materials_Analysis of PPARg signaling activity in psoriasis/Pathway models/Models images and html files/Differentiation of psoriatic T cells/6392.html]

IL18


# Protein IL18

|  |  |
| --- | --- |
| URN | urn:agi-llid:3606 |
| Total Entities | 0 |
| Connectivity | 5555 |
| Name | IL18 |
| Description | interleukin 18 |
| Notes | The protein encoded by this gene is a proinflammatory cytokine that augments natural killer cell activity in spleen cells, and stimulates interferon gamma production in T-helper type I cells. Alternatively spliced transcript variants encoding different isoforms have been found for this gene.[provided by RefSeq, Aug 2011] |
| Primary Cell Localization | Extracellular |
| Class | Ligand |
| ObjectType | Protein |

---

|  |  |
| --- | --- |
| Pathway | NOD-like Receptors |
|  | Th1-Cell Differentiation |
|  | Th17-Cell Differentiation |
|  | Toll-like Receptor Independent Sterile Inflammation |
|  | Lipids Enhance Apoptotic Cell Engulfment and Reduce Inflammation |
|  | Basophil Activation |
|  | Mast-Cells De Novo Synthesized Mediators via IgE Independent Signaling |
|  | Proteins Involved in Atherosclerosis |
|  | Th1-Cells Activation and Proliferation in Atherosclerosis |
|  | Proteins Involved in Myocardial Ischemia |
|  | Lymphocyte Mediated Myocardial Injury in Myocarditis |
|  | Proteins Involved in Myocarditis |
|  | Proteins Involved in Acne Vulgaris |
|  | Proteins Involved in Atopic Dermatitis |
|  | Proteins Involved in Psoriasis |
|  | T-Cells Differentiation Block in Psoriasis |
|  | Apoptotic Keratinocytes Clearance Recession in Systemic Lupus Erythematosus |
|  | Dendritic Cell Activation in Systemic Lupus Erythematosis |
|  | Proteins Involved in Systemic Lupus Erythematosus |
|  | Th17-Cell Function in Systemic Lupus Erythematosus |
|  | T-Cell Cytotoxic Response against Melanocytes in Vitiligo |
|  | Plasmacytoid Dendritic Cell in Diabetes Mellitus Type 1 |
|  | Proteins Involved in Diabetes Mellitus Type 1 |
|  | Conventional Dendritic Cell Role in Diabetes Mellitus Type 1 |
|  | Proteins Involved in Diabetic Nephropathy |
|  | Proteins Involved in Insulin Resistance |
|  | Proteins Involved in non-Alcoholic Fatty Liver Disease |
|  | Hashimoto's Thyroiditis |
|  | Proteins Involved in Obesity |
|  | CD4+T-Cell Response in Celiac Disease |
|  | Th1-Cell Activation in Crohn's Disease |
|  | Macrophage Dysfunction |
|  | Proteins Involved in Inflammatory Bowel Diseases |
|  | Proteins Involved in Helicobacter Infections |
|  | Dendritic Cell Function in Ulcerative Colitis |
|  | Antigen-Presenting Cell Role in Asthma |
|  | Th17-Cell Differentiation in Asthma |
|  | Proteins Involved in Osteoarthritis |
|  | Proteins Involved in Polycystic Ovary Syndrome |
|  | Dendritic Cell Dysfunction in Psoriatic Arthritis |
|  | Th17-Cell and Th1 Immune Responsein Psoriatic Arthritis |
|  | Synovial Fibroblast Activation by Citokines in Rheumatoid Arthritis |
|  | Proteins Involved in Rheumatoid Arthritis |
|  | Th17-Cell Numbers Reduction in HIV |
|  | Proteins Involved in Periodontitis |
|  | PAPA Syndrome |
|  | Mevalonate Kinase Deficiency |
|  | Cryopyrin (NLPR3) Associated Periodic Syndromes |
|  | Familial Mediterranean Fever |
|  | Proteins Involved in Glioma |
|  | Proteins Involved in Neuroblastoma |
|  | Proteins Involved in Multiple Sclerosis |
|  | Proteins Involved in Endometriosis |
|  | Proteins Involved in Spontaneous Abortion |
|  | Proteins Involved in Age-Related Macular Degeneration |
|  | Immune System Role in Otitis Media |
|  | Alveolar Macrophage Negative Regulation Declined in COPD |
|  | Alveolar Macrophages Dysfunction in COPD |
|  | Proteins Involved in Chronic Obstructive Pulmonary Disease |
|  | T-Cell Cytotoxic Activation |
|  | Proteins Involved in Pulmonary Emphysema |
|  | Proteins Involved in Glomerulonephritis |
|  | Asbestosis- and Silicosis-Associated Inflammation Provokes Cancer Development |
|  | Proteins with Altered Expression in Tumor-Promoting Inflammation |
|  | Proteins/Ions Transporters -> Expression Targets in Lymphoid System and Blood |
|  | GPCRs Family -> Expression Targets in Lymphoid System and Blood |
|  | Elevated Receptors -> Expression Targets in Thymus |
|  | P2RX7 -> Expression Targets in Nerve Tissue |
|  | TGFB1-TGFBR1 Expression Targets |
|  | e |
|  | pathway 4 |
|  | PPAR Psoriasis |
|  | pprarg neg, uknown targets, ps-positive |
|  | prarg neg,ukn expres targets, ps-positive |
|  | PPARG negative targets |
|  | pparg expr target\_regulators |
|  | Neighbors of disease exacerbation |
|  | Neighbors of tumor progression |
|  | Neighbors of cancer progression |
|  | Neighbors of inflammatory status |
|  | Neighbors of overall survival |
|  | Neighbors of disease severity |
|  | Neighbors of tumor size |
|  | Neighbors of lesion size |
|  | Neighbors of mortality |
|  | Neighbors of survival rate |
|  | Neighbors of infarct size |
|  | Neighbors of survival time |
|  | Neighbors of functional recovery |
|  | Neighbors of insulin sensitivity |
|  | Neighbors of bacterial load |
|  | Neighbors of graft survival |
|  | Neighbors of therapeutic efficacy |
|  | Neighbors of clinical stage |
|  | Neighbors of lethality |
|  | Neighbors of tumor regression |
|  | Neighbors of recurrence-free survival |
|  | Neighbors of disease-free survival |
|  | Neighbors of tumor response |
|  | Neighbors of treatment outcome |
|  | Neighbors of body weight |
|  | Neighbors of body weight gain |
|  | Neighbors of cardiovascular risk |
|  | Neighbors of microvessel density |
|  | Neighbors of tumor promotion |
|  | Neighbors of neutrophil count |
|  | Neighbors of glucose tolerance |
|  | Neighbors of morbidity |
|  | Neighbors of mortality rate |
|  | Neighbors of fat mass |
|  | Neighbors of adverse outcome |
|  | Neighbors of radioresistance |
|  | Neighbors of lesion area |
|  | Neighbors of progression-free survival |
|  | Neighbors of viral load |
|  | Neighbors of blood pressure |
|  | Neighbors of leukocyte count |
|  | Neighbors of vascular reactivity |
|  | Neighbors of vascular leakage |
|  | Neighbors of body mass index |
|  | Neighbors of tumor aggressiveness |
|  | Neighbors of systolic blood pressure |
|  | Neighbors of blood glucose |
|  | Neighbors of ejection fraction |
|  | Neighbors of median survival |
|  | Neighbors of fasting plasma glucose |
|  | Neighbors of T-cell count |
|  | Neighbors of antimetastatic activity |
|  | Neighbors of energy expenditure |
|  | Neighbors of vascular tone |
|  | Neighbors of breast cancer risk |
|  | Neighbors of functional status |
|  | Neighbors of neurological outcome |
|  | Neighbors of pathological response |
|  | Neighbors of lymphocyte count |
|  | Neighbors of monocyte count |
|  | Neighbors of comorbidity |
|  | Neighbors of carotid intima-media thickness |
|  | Neighbors of fibroblast phenotype |
|  | Neighbors of fractional shortening |
|  | Neighbors of disease exacerbation |
|  | Neighbors of tumor progression |
|  | Neighbors of cancer progression |
|  | Neighbors of inflammatory status |
|  | Neighbors of overall survival |
|  | Neighbors of disease severity |
|  | Neighbors of tumor size |
|  | Neighbors of lesion size |
|  | Neighbors of mortality |
|  | Neighbors of survival rate |
|  | Neighbors of infarct size |
|  | Neighbors of survival time |
|  | Neighbors of functional recovery |
|  | Neighbors of insulin sensitivity |
|  | Neighbors of bacterial load |
|  | Neighbors of graft survival |
|  | Neighbors of therapeutic efficacy |
|  | Neighbors of clinical stage |
|  | Neighbors of lethality |
|  | Neighbors of tumor regression |
|  | Neighbors of recurrence-free survival |
|  | Neighbors of disease-free survival |
|  | Neighbors of tumor response |
|  | Neighbors of treatment outcome |
|  | Neighbors of body weight |
|  | Neighbors of body weight gain |
|  | Neighbors of cardiovascular risk |
|  | Neighbors of microvessel density |
|  | Neighbors of tumor promotion |
|  | Neighbors of neutrophil count |
|  | Neighbors of glucose tolerance |
|  | Neighbors of morbidity |
|  | Neighbors of mortality rate |
|  | Neighbors of fat mass |
|  | Neighbors of adverse outcome |
|  | Neighbors of radioresistance |
|  | Neighbors of lesion area |
|  | Neighbors of progression-free survival |
|  | Neighbors of viral load |
|  | Neighbors of blood pressure |
|  | Neighbors of leukocyte count |
|  | Neighbors of vascular reactivity |
|  | Neighbors of vascular leakage |
|  | Neighbors of body mass index |
|  | Neighbors of tumor aggressiveness |
|  | Neighbors of systolic blood pressure |
|  | Neighbors of blood glucose |
|  | Neighbors of ejection fraction |
|  | Neighbors of median survival |
|  | Neighbors of fasting plasma glucose |
|  | Neighbors of T-cell count |
|  | Neighbors of antimetastatic activity |
|  | Neighbors of energy expenditure |
|  | Neighbors of vascular tone |
|  | Neighbors of breast cancer risk |
|  | Neighbors of functional status |
|  | Neighbors of neurological outcome |
|  | Neighbors of pathological response |
|  | Neighbors of lymphocyte count |
|  | Neighbors of monocyte count |
|  | Neighbors of comorbidity |
|  | Neighbors of carotid intima-media thickness |
|  | Neighbors of fibroblast phenotype |
|  | Neighbors of fractional shortening |
|  | Neighbors of cell development |
|  | Neighbors of immune response |
|  | Neighbors of cell population |
|  | Neighbors of T-cell development |
|  | Neighbors of inflammatory response |
|  | Neighbors of wound healing |
|  | Neighbors of angiogenesis |
|  | Neighbors of innate immune response |
|  | Neighbors of cell function |
|  | Neighbors of macrophage activation |
|  | Neighbors of cell formation |
|  | Neighbors of adaptive immune response |
|  | Neighbors of osteoclast development |
|  | Neighbors of epithelial to mesenchymal transition |
|  | Neighbors of immunity |
|  | Neighbors of cell proliferative response |
|  | Neighbors of chemotaxis |
|  | Neighbors of dendritic cell differentiation |
|  | Neighbors of cell survival |
|  | Neighbors of osteoclast differentiation |
|  | Neighbors of cell phenotype |
|  | Neighbors of T-cell proliferation |
|  | Neighbors of tissue remodeling |
|  | Neighbors of cell adhesion |
|  | Neighbors of T-cell activation |
|  | Neighbors of endothelial cell proliferation |
|  | Neighbors of tumor growth |
|  | Neighbors of dendritic cell development |
|  | Neighbors of tissue repair |
|  | Neighbors of cell count |
|  | Neighbors of cell infiltration |
|  | Neighbors of cell interaction |
|  | Neighbors of SMC proliferation |
|  | Neighbors of lumen formation |
|  | Neighbors of endothelial cell migration |
|  | Neighbors of adipogenesis |
|  | Neighbors of lipid storage |
|  | Neighbors of cell damage |
|  | Neighbors of pregnancy |
|  | Neighbors of monocyte differentiation |
|  | Neighbors of aging |
|  | Neighbors of cell motility |
|  | Neighbors of bone resorption |
|  | Neighbors of monocyte migration |
|  | Neighbors of fibroblast proliferation |
|  | Neighbors of macrophage migration |
|  | Neighbors of microglial activation |
|  | Neighbors of neutrophil recruitment |
|  | Neighbors of regeneration |
|  | Neighbors of cell migration |
|  | Neighbors of cancer growth |
|  | Neighbors of stem cell proliferation |
|  | Neighbors of smooth muscle cell migration |
|  | Neighbors of hepatic regeneration |
|  | Neighbors of transendothelial migration |
|  | Neighbors of phagocytosis |
|  | Neighbors of immune system activation |
|  | Neighbors of leukocyte migration |
|  | Neighbors of leukocyte recruitment |
|  | Neighbors of osteoclast formation |
|  | Neighbors of monocyte recruitment |
|  | Neighbors of cell invasion |
|  | Neighbors of heart function |
|  | Neighbors of bone remodeling |
|  | Neighbors of ossification |
|  | Neighbors of T-cell function |
|  | Neighbors of immune system function |
|  | Neighbors of lung development |
|  | Neighbors of autophagy |
|  | Neighbors of monocyte adhesion |
|  | Neighbors of senescence |
|  | Neighbors of T-cell response |
|  | Neighbors of tumor immunity |
|  | Neighbors of hepatocyte proliferation |
|  | Neighbors of cell transdifferentiation |
|  | Neighbors of stem cell differentiation |
|  | Neighbors of endothelial cell function |
|  | Neighbors of neuronal death |
|  | Neighbors of cell growth |
|  | Neighbors of ROS generation |
|  | Neighbors of epithelial cell proliferation |
|  | Neighbors of first trimester pregnancy |
|  | Neighbors of sensitization |
|  | Neighbors of hemato-encephalic barrier |
|  | Neighbors of macrophage function |
|  | Neighbors of cellular immune response |
|  | Neighbors of colony formation |
|  | Neighbors of cell differentiation |
|  | Neighbors of life span |
|  | Neighbors of cell homeostasis |
|  | Neighbors of psoriasis |
|  | Neighbors of inflammatory disease |
|  | Neighbors of rheumatoid arthritis |
|  | Neighbors of atherosclerosis |
|  | Neighbors of fibrosis |
|  | Neighbors of colitis |
|  | Neighbors of arthritis |
|  | Neighbors of inflammatory bowel disease |
|  | Neighbors of injury |
|  | Neighbors of atherogenesis |
|  | Neighbors of vascular remodeling |
|  | Neighbors of metastasis |
|  | Neighbors of carcinogenesis |
|  | Neighbors of multiple sclerosis |
|  | Neighbors of chronic inflammation |
|  | Neighbors of inflammation |
|  | Neighbors of tumor microenvironment |
|  | Neighbors of pathological angiogenesis |
|  | Neighbors of obesity |
|  | Neighbors of vascular disease |
|  | Neighbors of diabetes mellitus |
|  | Neighbors of asthma |
|  | Neighbors of pneumonia |
|  | Neighbors of hyperplasia |
|  | Neighbors of osteoarthritis |
|  | Neighbors of liver fibrosis |
|  | Neighbors of chronic obstructive pulmonary disease |
|  | Neighbors of myocardial infarction |
|  | Neighbors of leukocyte infiltration |
|  | Neighbors of reperfusion injury |
|  | Neighbors of airway inflammation |
|  | Neighbors of hepatitis |
|  | Neighbors of insulin resistance |
|  | Neighbors of infection |
|  | Neighbors of vascular injury |
|  | Neighbors of renal injury |
|  | Neighbors of preeclampsia |
|  | Neighbors of macrophage infiltration |
|  | Neighbors of liver injury |
|  | Neighbors of neuroinflammation |
|  | Neighbors of sepsis |
|  | Neighbors of death |
|  | Neighbors of type 1 diabetes |
|  | Neighbors of encephalomyelitis |
|  | Neighbors of autoimmune disease |
|  | Neighbors of ischemic stroke |
|  | Neighbors of dermatitis |
|  | Neighbors of neutrophil infiltration |
|  | Neighbors of heart disease |
|  | Neighbors of diabetic nephropathy |
|  | Neighbors of lung injury |
|  | Neighbors of immunopathology |
|  | Neighbors of systemic sclerosis |
|  | Neighbors of hepatocellular carcinoma |
|  | Neighbors of autoimmunity |
|  | Neighbors of acute lung injury |
|  | Neighbors of gastric cancer |
|  | Neighbors of atherosclerotic plaque |
|  | Neighbors of experimental autoimmune encephalomyelitis |
|  | Neighbors of colorectal cancer |
|  | Neighbors of cardiac remodeling |
|  | Neighbors of breast cancer |
|  | Neighbors of ischemia |
|  | Neighbors of systemic lupus erythematosus |
|  | Neighbors of melanoma |
|  | Neighbors of collagen-induced arthritis |
|  | Neighbors of cartilage degeneration |
|  | Neighbors of interstitial fibrosis |
|  | Neighbors of neoplasm invasion |
|  | Neighbors of kidney disease |
|  | Neighbors of heart failure |
|  | Neighbors of lung disease |
|  | Neighbors of pulmonary fibrosis |
|  | Neighbors of cardiovascular disease |
|  | Neighbors of stroke |
|  | Neighbors of cancer |
|  | Neighbors of cerebral ischemia |
|  | Neighbors of type 2 diabetes |
|  | Neighbors of neoplasm |
|  | Neighbors of endometriosis |
|  | Neighbors of neointima |
|  | Neighbors of vasculitis |
|  | Neighbors of atrophy |
|  | Neighbors of diet-induced obesity |
|  | Neighbors of respiratory hypersensitivity |
|  | Neighbors of myocardial fibrosis |
|  | Neighbors of synovitis |
|  | Neighbors of coronary artery disease |
|  | Neighbors of rupture |
|  | Neighbors of endothelial cell dysfunction |
|  | Neighbors of liver disease |
|  | Neighbors of prostate cancer |
|  | Neighbors of hypertrophy |
|  | Neighbors of osteolysis |
|  | Neighbors of ovarian cancer |
|  | Neighbors of pancreatic cancer |
|  | Neighbors of hypertension |
|  | Neighbors of intimal hyperplasia |
|  | Neighbors of allograft rejection |
|  | Neighbors of proteinuria |
|  | Neighbors of epithelial cell |
|  | Neighbors of keratinocyte |
|  | Neighbors of vascular smooth muscle cell |
|  | Neighbors of umbilical vein endothelial cell |
|  | Neighbors of monocyte |
|  | Neighbors of alveolar macrophage |
|  | Neighbors of synoviocyte |
|  | Neighbors of macrophage |
|  | Neighbors of adipocyte |
|  | Neighbors of activated monocyte |
|  | Neighbors of fibroblast |
|  | Neighbors of airway epithelial cell |
|  | Neighbors of PBMC |
|  | Neighbors of stromal cell |
|  | Neighbors of osteoblast |
|  | Neighbors of pneumocyte |
|  | Neighbors of endometrium cell |
|  | Neighbors of microglia |
|  | Neighbors of trophoblast |
|  | Neighbors of intestine epithelium cell |
|  | Neighbors of mononuclear cell |
|  | Neighbors of hepatocyte |
|  | Neighbors of kidney mesangial cell |
|  | Neighbors of endothelial cell |
|  | Neighbors of dendritic cell |
|  | Neighbors of Kuppfer cell |
|  | Neighbors of proadipocyte |
|  | Neighbors of kidney tubule cell |
|  | Neighbors of inflammatory cell |
|  | Neighbors of bone marrow macrophage |
|  | Neighbors of peritoneal macrophage |
|  | Neighbors of osteoclast |
|  | Neighbors of neutrophil |
|  | Neighbors of smooth muscle myocyte |
|  | Neighbors of astrocyte |
|  | Neighbors of bone marrow derived dendritic cell |
|  | Neighbors of activated T-cell |
|  | Neighbors of mesothelial cell |
|  | Neighbors of mononuclear phagocyte |
|  | Neighbors of immunocompetent cell |
|  | Neighbors of granulocyte |
|  | Neighbors of cardiac myocyte |
|  | Neighbors of myeloid cell |
|  | Neighbors of mesenchymal stroma cell |
|  | Neighbors of stem cell |
|  | Neighbors of tumor infiltrating macrophage |
|  | Neighbors of sinusoidal cell |
|  | Neighbors of B-cell |
|  | Neighbors of leukocyte |
|  | Neighbors of chondrocyte |
|  | Neighbors of insulin-secreting cell |
|  | Neighbors of T-cell |
|  | Neighbors of M1 macrophage |
|  | Neighbors of mast cell |
|  | Neighbors of spleen cell |
|  | Neighbors of neuroglia |
|  | Neighbors of hematopoietic cell |
|  | Neighbors of adipose macrophage |
|  | Neighbors of podocyte |
|  | Neighbors of mammary epithelial cell |
|  | Neighbors of conventional dendritic cell |
|  | Neighbors of antigen-presenting cell |
|  | Neighbors of epidermis cell |
|  | Neighbors of phagocyte |
|  | Neighbors of kidney cell |
|  | Neighbors of lymphocyte |
|  | Neighbors of Langerhans cell |
|  | Neighbors of thymic epithelial cell |
|  | Neighbors of brain cell |
|  | Neighbors of M2 macrophage |
|  | Neighbors of peritoneum cell |
|  | Neighbors of lymphoid cell |
|  | Neighbors of blood cell |
|  | Neighbors of skin cell |
|  | Neighbors of lymph node cell |
|  | Neighbors of lung |
|  | Neighbors of liver |
|  | Neighbors of kidney |
|  | Neighbors of skin |
|  | Neighbors of intestine |
|  | Neighbors of lymph node |
|  | Neighbors of colon |
|  | Neighbors of airway |
|  | Neighbors of blood vessel |
|  | Neighbors of heart |
|  | Neighbors of aorta |
|  | Neighbors of gastrointestinal tract |
|  | Neighbors of bone |
|  | Neighbors of placenta |
|  | Neighbors of carotid artery |
|  | Neighbors of synovial membrane |
|  | Neighbors of esophagus |
|  | Neighbors of periodontium |
|  | Neighbors of retina |
|  | Neighbors of brain |
|  | Neighbors of breast |
|  | Neighbors of blood vessel wall |
|  | Neighbors of stomach |
|  | Neighbors of islets of Langerhans |
|  | Neighbors of cardiovascular system |
|  | Neighbors of pancreas |
|  | Neighbors of prostate |
|  | Neighbors of central nervous system |
|  | Neighbors of kidney glomerulus |
|  | Neighbors of immune system |
|  | Neighbors of spleen |
|  | Neighbors of peritoneum |
|  | Neighbors of left ventricle |
|  | Neighbors of omentum |
|  | Neighbors of vein |
|  | Neighbors of glands |
|  | Neighbors of hippocampus |
|  | Neighbors of cerebral cortex |
|  | Neighbors of nervous system |
|  | Neighbors of nasopharynx |
|  | Neighbors of salivary gland |
|  | Neighbors of ileum |
|  | Neighbors of aggregated lymphoid follicle of small intestine |
|  | Neighbors of NF-kB family |
|  | Neighbors of oxidized LDL |
|  | Neighbors of JNK |
|  | Neighbors of PPAR |
|  | Neighbors of Jun/Fos |
|  | Neighbors of PKC |
|  | Neighbors of PI3K |
|  | Neighbors of STAT family |
|  | Neighbors of mitogen-activated protein kinase |
|  | Neighbors of IFNAR ligand |
|  | Neighbors of TLR |
|  | Neighbors of JAK |
|  | Neighbors of PKA |
|  | Neighbors of beta adrenoceptor |
|  | Neighbors of IKBK |
|  | Neighbors of adipokine |
|  | Neighbors of IgG |
|  | Neighbors of LXR |
|  | Neighbors of p38 |
|  | Neighbors of IL23 |
|  | Neighbors of PDGF-BB |
|  | Neighbors of IL12 |
|  | Neighbors of prostaglandin-endoperoxide synthase |
|  | Neighbors of heat shock protein 90 |
|  | Neighbors of lectin |
|  | Neighbors of Ang II receptor |
|  | Neighbors of superoxide dismutase |
|  | Neighbors of proteasome endopeptidase complex |
|  | Neighbors of class nuclear receptor with C4 zinc fingers |
|  | Neighbors of WNT |
|  | Neighbors of immunoglobulin |
|  | Neighbors of PP2B |
|  | Neighbors of ITG |
|  | Neighbors of relaxin |
|  | Neighbors of histone |
|  | Neighbors of Rho kinase |
|  | Neighbors of caspase |
|  | Model of PPARG signaling in psoriasis |
|  | PPARG negative regulators and targets |
|  | Model of PPARG related pathways in psoriasis (short version) |
|  | dsRNA induces innate immunity and INFs in alveolar cells |
|  | Dendritic Cell Activation in COVID19 |
|  | Overview. Diseases of the skin (chapter 11) |
|  | 1\_Differentiation of psoriatic T cells |
|  | 1\_Insufficient activation of immune response in the middle ear epithelium cells in otitis media |
|  | 3\_Role of alveolar macrophages in pulmonary tissue destruction in COPD |
|  | 1\_Th2 cell response in asthma |
|  | 1\_Defects in response to pathogens in the gut promotes inflammation in Crohn's disease |
|  | 2\_Intestine epithelial cells drive immunological responses in ulcerative colitis |
|  | 4\_1\_Polymorphisms associated with inflammatory bowel diseases |
|  | 2\_Lipotoxicity in NAFLD |
|  | 1\_Antigen-presenting cells promote islet dysfunction and immune system activation in T1D |
|  | 2\_Defective tolerance of autoreactive T-cell in T1D |
|  | Figure 4\_Diabetes mellitus Type 1: conventional dendritic cell |
|  | drug-target moltransport |
|  | Differentiation of psoriatic T cells |

---

|  |  |
| --- | --- |
| Group | Genes Associated with Systemic Lupus Erythematosus |

---

|  |  |
| --- | --- |
| MedScan ID | 3606 |

---

|  |  |
| --- | --- |
| LocusLink ID | 3606 |
|  | 16173 |
|  | 29197 |

---

|  |  |
| --- | --- |
| Alias | interleukin IL18\_HUMAN |
|  | IFN-gamma-inducing factor |
|  | Interleukin-1 gamma |
|  | IL-1g |
|  | interleukin 18 |
|  | Interferon-gamma inducing factor |
|  | IL18 |
|  | IL1F4 |
|  | MGC12320 |
|  | interleukin IL18 |
|  | IL-1 gamma |
|  | IL18\_HUMAN |
|  | interleukin 18 (interferon-gamma-inducing factor) |
|  | gamma interferon inducing factor |
|  | interleukin IL1F4 |
|  | IGIF |
|  | interleukin IL-1 gamma |
|  | interleukin IL-1g |
|  | IL-18 |
|  | iboctadekin |
|  | interleukin-18 |
|  | interferon gamma-inducing factor |
|  | interferon-gamma-inducing factor |
|  | interleukin IL-18) (INTERFERON-GAMMA INDUCING FACTOR |
|  | IL-18) (INTERFERON-GAMMA INDUCING FACTOR |

---

|  |  |
| --- | --- |
| GO ID | 0005125 |
|  | 0045515 |
|  | 0000165 |
|  | 0035744 |
|  | 0042088 |
|  | 0032148 |
|  | 0001525 |
|  | 0008283 |
|  | 0007267 |
|  | 0071407 |
|  | 0042033 |
|  | 0042632 |
|  | 0019221 |
|  | 0042253 |
|  | 0006954 |
|  | 0042095 |
|  | 0042231 |
|  | 0035655 |
|  | 0042094 |
|  | 0032635 |
|  | 0031663 |
|  | 0030101 |
|  | 0042267 |
|  | 0045662 |
|  | 0042119 |
|  | 0051092 |
|  | 1901224 |
|  | 0051142 |
|  | 2000556 |
|  | 0045630 |
|  | 0042104 |
|  | 0120162 |
|  | 0032725 |
|  | 0050729 |
|  | 0032729 |
|  | 0032740 |
|  | 0010744 |
|  | 0032819 |
|  | 0150078 |
|  | 0014068 |
|  | 0051897 |
|  | 0048661 |
|  | 0034105 |
|  | 0045944 |
|  | 0042531 |
|  | 0030155 |
|  | 0030431 |
|  | 0070328 |
|  | 0042092 |
|  | 0005829 |
|  | 0005576 |
|  | 0005615 |
|  | 0071375 |
|  | 0050966 |
|  | 0008625 |
|  | 0006955 |
|  | 0033030 |
|  | 0050732 |
|  | 0043065 |
|  | 2000504 |
|  | 0032722 |
|  | 0032967 |
|  | 0010628 |
|  | 0032757 |
|  | 2000256 |
|  | 0043525 |
|  | 0014911 |
|  | 0032930 |
|  | 0032760 |
|  | 0043117 |
|  | 0001666 |
|  | 0016324 |
|  | 0005737 |
|  | 0071320 |
|  | 0070301 |
|  | 0071346 |
|  | 0071222 |
|  | 0071260 |
|  | 0071374 |
|  | 0071356 |
|  | 0048546 |
|  | 0032609 |
|  | 0007611 |
|  | 0030324 |
|  | 0001649 |
|  | 0008284 |
|  | 0032731 |
|  | 0045188 |
|  | 0010469 |
|  | 0009409 |
|  | 0055093 |
|  | 0043434 |
|  | 0009314 |
|  | 0005622 |
|  | 0070062 |
|  | 0060312 |
|  | 0042346 |
|  | 0042517 |
|  | 0004871 |
|  | 0007165 |

---

|  |  |
| --- | --- |
| KEGG ID | hsa:3606 |
|  | mmu:16173 |
|  | rno:29197 |

---

|  |  |
| --- | --- |
| Organism | Homo sapiens {Organism urn:agi-taxid:9606} |
|  | Mus musculus {Organism urn:agi-taxid:10090} |
|  | Rattus norvegicus {Organism urn:agi-taxid:10116} |
|  | Homo sapiens |
|  | Mus musculus |
|  | Rattus norvegicus |

---

|  |  |
| --- | --- |
| Mouse chromosome position | 9 27.75 cM |
|  | 9 29.0 cM |

---

|  |  |
| --- | --- |
| OMIM ID | 600953 |

---

|  |  |
| --- | --- |
| Rat chromosome position | 8q23 |

---

|  |  |
| --- | --- |
| Hugo ID | 5986 |
|  | HGNC:5986 |

---

|  |  |
| --- | --- |
| Human chromosome position | 11q23.1 |
|  | 11q22.2-q22.3 |

---

|  |  |
| --- | --- |
| Swiss-Prot Accession | A0A024R3E0 |
|  | Q14116 |
|  | Q14116.1 |
|  | P70380 |
|  | Q2PMY2 |
|  | P70380.2 |
|  | P97636 |
|  | P97636.1 |
|  | O75599 |
|  | Q6FGY3 |
|  | Q6WWJ7 |
|  | O88749 |
|  | P97637 |
|  | Q541E6 |
|  | Q96KJ8 |
|  | Q9NQ49 |
|  | Q6USQ6 |
|  | Q80SS8 |

---

|  |  |
| --- | --- |
| PIR ID | S60226 |

---

|  |  |
| --- | --- |
| GenBank ID | NC\_000011 |
|  | XM\_011542805 |
|  | XP\_011541107 |
|  | XM\_011542806 |
|  | XP\_011541108 |
|  | NM\_001243211 |
|  | NP\_001230140 |
|  | NM\_001562 |
|  | NP\_001553 |
|  | NG\_028143 |
|  | AB015961 |
|  | AB095532 |
|  | AJ295724 |
|  | CAC01436 |
|  | AP002007 |
|  | AP002884 |
|  | CH471065 |
|  | EAW67184 |
|  | EAW67185 |
|  | EF444989 |
|  | ACA06006 |
|  | AF077611 |
|  | AAC27787 |
|  | AF380360 |
|  | AAK57024 |
|  | AK311301 |
|  | AY044641 |
|  | AAK95950 |
|  | AY266351 |
|  | AAP92112 |
|  | BC007007 |
|  | AAH07007 |
|  | BC007461 |
|  | AAH07461 |
|  | BC015863 |
|  | BU633686 |
|  | CR541973 |
|  | CAG46771 |
|  | CR542001 |
|  | CAG46798 |
|  | D49950 |
|  | BAA08706 |
|  | U90434 |
|  | AAB50010 |
|  | Q14116 |
|  | NC\_000075 |
|  | XM\_006510024 |
|  | XP\_006510087 |
|  | XM\_006510026 |
|  | XP\_006510089 |
|  | XM\_006510027 |
|  | XP\_006510090 |
|  | XM\_017313137 |
|  | XP\_017168626 |
|  | XM\_006510028 |
|  | XP\_006510091 |
|  | NM\_008360 |
|  | NP\_032386 |
|  | NM\_001357221 |
|  | NM\_001357222 |
|  | AC025500 |
|  | AC127254 |
|  | AJ002364 |
|  | CH466522 |
|  | EDL25740 |
|  | AK078100 |
|  | AV328761 |
|  | AY157834 |
|  | AAO21309 |
|  | AY157835 |
|  | AAO21310 |
|  | AY362457 |
|  | AAQ63045 |
|  | AY916131 |
|  | AAY16325 |
|  | BC024384 |
|  | AAH24384 |
|  | CX123286 |
|  | D49949 |
|  | BAA08705 |
|  | DQ316604 |
|  | ABC43200 |
|  | KY491641 |
|  | AQX45480 |
|  | L37150 |
|  | U66244 |
|  | AAB49753 |
|  | P70380 |
|  | NC\_005107 |
|  | NM\_019165 |
|  | NP\_062038 |
|  | AC\_000076 |
|  | AAHX01054600 |
|  | AAHX01054601 |
|  | AC141541 |
|  | CH473975 |
|  | EDL95461 |
|  | Y13337 |
|  | CAA73781 |
|  | AJ222813 |
|  | CAA11001 |
|  | AY077842 |
|  | AAL77211 |
|  | AY258448 |
|  | AAP14669 |
|  | U77776 |
|  | AAC53009 |
|  | U77777 |
|  | AAC53010 |
|  | P97636 |
|  | AABR06054732 |
|  | XM\_006510025 |
|  | ABBA01039802 |
|  | ABBA01039803 |
|  | XM\_006510023 |
|  | ABBA01039804 |
|  | XP\_011240704 |
|  | XP\_011240703 |
|  | XP\_006510086 |
|  | XP\_006510088 |
|  | AC\_000031 |
|  | AC\_000143 |
|  | NC\_018922 |
|  | AAHY01078036 |
|  | AAHY01078037 |
|  | AMYH02025585 |
|  | XM\_011242401 |
|  | XM\_011242402 |
|  | NT\_033899 |
|  | NW\_001838042 |
|  | NW\_925173 |
|  | AC\_000054 |
|  | Q6WWJ7 |
|  | Q96KJ8 |
|  | Q9NQ49 |
|  | NT\_039472 |
|  | NW\_001030907 |
|  | Q2PMY2 |
|  | Q6USQ6 |
|  | Q80SS8 |
|  | NW\_047799 |
|  | NW\_001084873 |

---

|  |  |
| --- | --- |
| Swiss-Prot ID | IL18\_HUMAN |
|  | IL18\_MOUSE |
|  | IL18\_RAT |
|  | Q2PMY2\_MOUSE |

---

|  |  |
| --- | --- |
| Cell Localization | Secreted |
|  | Extracellular |

---

|  |  |
| --- | --- |
| Ensembl ID | ENSG00000150782 |
|  | ENSP00000434561.1 |
|  | ENST00000524595.5 |
|  | ENSP00000280357.7 |
|  | ENST00000280357.12 |
|  | ENSP00000434161.1 |
|  | ENST00000528832.1 |
|  | ENSMUSG00000039217 |
|  | ENSMUSP00000151002.1 |
|  | ENSMUST00000214117.1 |
|  | ENSRNOG00000009848 |
|  | ENSRNOP00000013093.6 |
|  | ENSRNOT00000013093.6 |
|  | ENST00000280357.11 |
|  | ENST00000524595 |
|  | ENST00000528832 |
|  | ENSP00000280357 |
|  | ENST00000280357 |
|  | ENSMUST00000180021 |
|  | ENSMUSP00000137193 |
|  | ENSP00000434161 |
|  | ENSRNOT00000013093 |
|  | ENSP00000434561 |
|  | ENSRNOP00000013093 |

---

|  |  |
| --- | --- |
| MGI ID | MGI:107936 |
|  | 107936 |

---

|  |  |
| --- | --- |
| RGD ID | 2889 |

---

|  |  |
| --- | --- |
| Unigene ID | Rn.11118 |
|  | Hs.83077 |
|  | Mm.1410 |

---

|  |  |
| --- | --- |
| Homologene ID | 1200 |

---

|  |  |
| --- | --- |
| Shape | Rhomb |

---

|  |  |
| --- | --- |
| IPI ID | IPI00206468 |
|  | IPI00290198 |
|  | IPI00307902 |
|  | IPI00231939 |

---
